# “Freely explore this environment”: individual differences in exploration behavior and survey knowledge

**DOI:** 10.1186/s41235-025-00696-5

**Published:** 2025-12-19

**Authors:** Veronica Muffato, Laura Miola, Sara Errigo, Francesca Pazzaglia, Chiara Meneghetti

**Affiliations:** 1 Department of General Psychology, University of Padova, Via Venezia 8, 35131 Padua, Italy; 2 Interuniversity Research Centre in Environmental Psychology (CIRPA), Rome, Italy

**Keywords:** Exploration, Navigation, Visuospatial working memory, Spatial self-efficacy, Survey knowledge

## Abstract

When navigating individuals form mental representations of their environment. Significant individual differences exist, particularly in the ability to develop survey knowledge—a map-like understanding of spatial relationships. While many studies have focused on navigation via predetermined routes, less is known about individual differences in learning environments from free exploration. This study investigated whether exploration behaviors, such as the tendency to revisit places or diffuse in the environment, are linked to mental representations and whether visuospatial factors, like visuospatial working memory and self-reported wayfinding inclinations (e.g., self-efficacy and pleasure in exploring, and spatial anxiety), contribute to these relationships. A sample of 234 participants (147 womencompleted a visuospatial working memory task and wayfinding questionnaires, then freely explored a virtual city in a CAVE environment. Revisiting (tendency to re-explore visited areas) and diffusion (extent of environmental coverage) were calculated. Their survey knowledge was assessed using a map drawing task. The results showed that exploration behaviors were related to individual differences: women exhibited less revisiting and diffusion than men; higher self-efficacy and pleasure in exploring were associated with reduced revisiting; and joystick familiarity favoured diffusion. Furthermore, exploration behaviors mediated the relationship between gender and self-efficacy and pleasure in exploring with map accuracy. Map drawing was also directly predicted by visuospatial working memory ability. These findings offer insights into the role of individual differences in exploration behaviors and their relationship to survey knowledge gained from free exploration, highlighting the importance of individual factors in how environments are experienced and processed.

**Significance statement**

Some people are better at understanding where landmarks are in a place and how they are connected, while others find it more difficult. This paper investigates whether the way we move through an environment (how we explore) relates to this ability, along with other personal factors. When exploring a city, some people move around a lot, while others stay closer to familiar areas. We found that this is related to how well people understand the layout of the place. Importantly, we also found that personal attitudes toward orientation matter: people who feel more confident and enjoy exploring tend to revisit places less. On the other hand, the ability to mentally handle visual and spatial information (visuospatial working memory) doesn’t change how we explore, but it does help us build a better understanding of the environment. Gender and familiarity with using a joystick also related to how people explored and what they learned. By studying how people move and how this relates to their individual characteristics, we can better understand how we learn about the environments.

**Supplementary Information:**

The online version contains supplementary material available at 10.1186/s41235-025-00696-5.

Navigating through environments allows individuals to form environmental mental representations (Wolbers & Hegarty, 2010; or cognitive maps, Tolman, 1948), which flexibly store knowledge about landmarks, routes, and the relationships between them—referred to as survey knowledge. Through navigation, individuals learn about their environment from their point of view (egocentric system). Over time, this knowledge can detach from the learner's perspective and evolve into a more global perspective, independent of the learner's viewpoint (survey knowledge, which can refer to the allocentric system). Building survey knowledge is central to accomplishing environmental tasks and developing a refined understanding of spatial relationships (Wiener et al., 2009). The task used to assess this type of knowledge are, for instance, pointing and map tasks (Hegarty et al., 2006). They effectively evaluate navigation ability because they require individuals to manage and transform spatial information from the perspective used during the learning phase (egocentric) to a map-like perspective (allocentric) in the testing phase (Muffato et al., 2025).

However, the ability to form survey knowledge is related to great individual differences (e.g., Hegarty et al., 2006; Muffato et al., 2020; Weisberg et al., 2014; Zhong & Kozhevnikov, 2016). Among the key factor in forming survey knowledge, there is an individual’s visuo-spatial working memory (e.g., Weisberg et al., 2014), i.e., the ability to temporarily store and manipulate visual and spatial information, and higher order visuospatial abilities, such as the ability to mentally rotate and take other perspectives (Hegarty et al., 2006). Non-cognitive factors also appear to be related to the ability to form survey knowledge (Muffato et al., 2022). They include, for instance, a set of wayfinding inclinations such as self-reported sense of direction, spatial self-efficacy, attitudes toward navigation tasks, and spatial anxiety. This suggests that self-reported, emotional and motivational factors may play a role in the formation of an environmental mental representation (Miola et al., 2021; Muffato et al., 2025).

Most studies in this field have focused on individual differences and their relationships to mental representations of the environment when asking participants to navigate predetermined paths. These paths were navigated using cues such as avatars, instructions, or arrows, either passively (e.g., being transported, as in video learning) or actively (e.g., self-directed movement along the path; Chrastil & Warren, 2015). On the contrary, the study of free exploration in environments, where individuals can freely navigate and choose where to go, and how the patterns of exploration behavior associate to the formation of environmental mental representations have been less investigated. Some studies have explored this topic, typically evaluating exploration behavior using various indices that assess how individuals navigate within an environment. Among these indexes, path length is commonly used to measure the extent of spatial coverage, along with other navigational behaviors that reflect decision-making during exploration, such as the number of turns and pauses (e.g., Chrastil & Warren, 2013, 2014; Farran et al., 2022; Puthusseryppady et al., 2024). More refined variables can also be calculated, such as revisiting behavior that measures how often participants return to previously explored areas (Farran et al., 2022; Gagnon et al., 2016, 2018; Munion et al., 2019) and the diffusion index, which is a metric quantifying how widely and uniformly exploration is distributed across the environment (Gagnon et al., 2018). Similarly, roaming Shannon’ entropy can be calculated (Brunec et al., 2023; Puthusseryppady et al., 2024; Walkowiak et al., 2015) as a measure of the probability distribution of a participant's location within an environment during exploration, quantifying the spread and evenness of their movement behavior. Some studies have found that certain exploration behaviors support the formation of mental representations of the environment. For example, lower revisiting behavior and higher diffusion behavior have been associated with successful navigation and reduced pointing errors (Farran et al., 2022; Gagnon et al., 2016, 2018). In contrast, other studies have found no significant relationship between exploration patterns (e.g., entropy) and spatial knowledge (e.g., Brunec et al., 2023). It should be noted that most of these studies focused on navigation success, defined as the ability to find target locations and take the shortest paths to reach them (e.g., Chrastil & Warren, 2013, 2014; Farran et al., 2022; Gagnon et al., 2016, 2018; Munion et al., 2019; Puthusseryppady et al., 2024; Sutton et al., 2014; Wiener & Mallot, 2003). Additionally, some of these studies investigated the pointing task, which assesses allocentric knowledge of landmarks but also involves egocentric perspective-taking (e.g., Brunec et al., 2023; Gagnon et al., 2016, 2018; Keller & Sutton, 2022; Walter et al., 2022). Less is known about survey knowledge of the environment as assessed by map tasks (e.g., Brunec et al., 2023; Cen et al., 2024; Gehrke et al., 2018; Keller & Sutton, 2022). Some studies measured performance on map tasks but did not relate it to exploration behaviors (e.g., Keller & Sutton, 2022). Others showed that better map task performance was associated with exploring important areas of the environment (i.e., highly integrated regions; Brunec et al., 2023), with shorter exploration length (Gehrke et al., 2018) and with higher roaming entropy (Cen et al., 2024). However, a deeper understanding of survey knowledge acquisition in relation to exploration behavior indices is still needed.

Another important aspect that warrants further investigation is the role of individual differences in relating to exploration behaviors and, in turn, to the survey knowledge. Gender has been so far the most commonly studied individual difference factor in this area. Men tend to exhibit longer path lengths, fewer pauses, higher diffusion, and lower revisiting behavior compared to women (Farran et al., 2022; Gagnon et al., 2016, 2018; Munion et al., 2019). Munion et al. (2019) specifically suggested that the relationship between gender and navigational success (i.e., the ability to find targets in environments with a map) is fully mediated by exploration patterns. These findings align with evidence that women tend to exhibit greater spatial anxiety, leading to safer and more controlled exploration strategies (Lawton, 1994). However, less is known about how gender differences in exploration impact the acquisition of survey knowledge.

Regarding other individual differences, such as visuospatial abilities and self-reported wayfinding inclinations, most studies did not examine its association with exploration patterns. In studies that explored this relationship, a higher sense of direction was associated with increased revisiting, fewer pauses, and greater directional persistence (e.g., Munion et al., 2019), while another study found no significant correlation (e.g., Brunec et al., 2023). Similarly, a study found a relationship between higher spatial anxiety and increased revisiting as well as lower diffusion (Gagnon et al., 2018). However, to our knowledge, no studies have investigated the relationship between visuospatial working memory and exploration behavior. Although visuospatial working memory is known to play a relevant role in navigation and survey knowledge (Muffato et al., 2020; Weisberg et al., 2014), it is not yet clear whether it also relate to exploration patterns – a novel issue that the present study aims to address. Similarly, research has shown that both spatial self-efficacy (e.g., Miola et al., 2021) and the pleasure of exploring (Muffato & De Beni, 2020) are attitudes toward orientation tasks related to navigation and the acquisition of survey knowledge. However, no study has examined self-reported measures of self-efficacy and pleasure specifically in relation to everyday exploration, and how these relate to actual exploratory behaviors in the environment – a novel issue that the present study aims to investigate.

Based on these premises, the present study aimed to investigate the role of individual differences in exploration behaviors and their consequent impact on survey knowledge. We considered both cognitive aspects, such as visuospatial working memory, and self-reported wayfinding inclinations, such as self-efficacy and pleasure in exploring, and spatial anxiety. These factors are all known to play an important role in the formation of survey knowledge, yet they have not been jointly examined in relation to exploration patterns. See the theoretical model investigated in Fig. 1. Specifically, we investigated the direct effects of individual differences on both exploration behaviors and the survey knowledge gained from free exploration. Additionally, we investigated the possible mediating role of exploration behaviors in the relationship between individual differences and survey knowledge. Individual differences, such as gender, age, and joystick familiarity, will also be taken into account due to their potential role (e.g., Chrastil & Warren, 2014; Gagnon et al., 2018; Puthusseryppady et al., 2024; Ward et al., 2025).

**Fig. 1:**
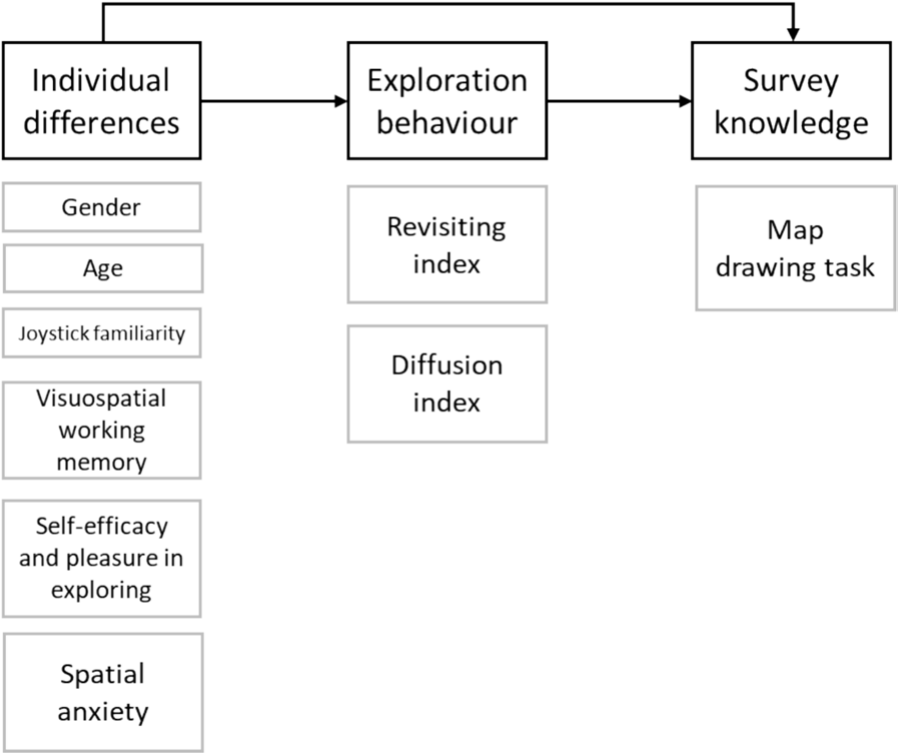
The hypothesized theoretical model

Concerning our hypothesis, we expected:

- A direct relationship between individual differences and exploration behavior. In particular, higher self-efficacy and pleasure in exploring is expected to be associated with higher diffusion and lower revisiting in the virtual environment (e.g., Muffato & De Beni, 2020; Munion et al., 2019). Spatial anxiety is also expected to be related to exploration measures (Gagnon et al., 2018), where higher spatial anxiety might be associated with higher revisiting and lower diffusion. The potential direct effect of visuospatial working memory on exploration behaviors will also be investigated, as no previous study has examined this.
- A direct relationship between individual differences and survey knowledge, as measured by the map drawing task. Visuospatial working memory is expected to be related to map drawing accuracy, as after leaning predetermined paths (e.g., Labate et al., 2014; Muffato et al., 2025). Self-efficacy and pleasure in exploring may not be directly related to map drawing task, as this measure primarily captures habits of exploration rather than spatial cognitive outcomes. Spatial anxiety, on the other hand, may or may not be related to survey knowledge, as previous research has reported both findings (e.g., Gagnon et al., 2018; Geer et al., 2024).
- Indirect relationships are also explored. Specifically, we expect that exploration behavior could mediate the relationship between self-efficacy and pleasure in exploring places and survey knowledge. This is plausible, given that habitual confidence and enjoyment in exploration could encourage more effective exploration behaviors, which, in turn, may support survey knowledge. Other potential indirect effects will also be explored. A mediating role of exploration behavior is plausible, as Munion et al. (2019) showed that exploration behavior can mediate the relationship between individual differences—in their case gender—and spatial knowledge—in their case route knowledge.

Concerning gender, we expected women showing greater revisiting and lower diffusion indices than men (e.g., Gagnon et al., 2018). We may or may not find a direct relationship between gender and survey knowledge, similar to results from learning via predetermined paths (e.g., Nazareth et al., 2019). Furthermore, we expected exploration behaviors to mediate the relationship between gender and survey knowledge, consistent with findings on route knowledge (e.g., navigation success; Munion et al., 2019). In terms of joystick familiarity, we expected an impact on exploration behaviors (Chrastil & Warren, 2014; Puthusseryppady et al., 2024).

## Method

### Participants

The study involved 234 young adults (147 women, 87 men, none “other/prefer not to answer”) from 19 to 36 years old (women *M age* = 20.77, *SD* = 1.48; men: *M age* = 21.70, *SD* = 2.29) recruited from two university courses in exchange for course credit and by word of mouth. The inclusion criteria were the following. Italian mother tongue, 18–40 years old, no self-reported motion sickness. Exclusion criteria were a history of psychiatric, neurological, or diseases capable of causing cognitive, visual, auditory, and/or motor impairments, incomplete data between sessions 1 and 2, technical problems during session 2. Originally, we enrolled 248 participants and we excluded 13 of them: 5 for reporting diseases, 5 for not completing session 1, and 3 for technical issues.

The sample size was determined by considering five to ten observations for each parameter estimated in the model (Bollen, 1989). Our model has 24 parameters (see Results section), so a sample size of 120–240 participants was considered sufficient. Furthermore, following the inverse square root method, a sample size of 233 participants suffices considering the minimum absolute value of the statistically significant path coefficient set at 0.16 (Kock & Hadaya, 2018).

The Ethics Committee for Psychological Research at University of Padova approved the study (No. 209-b). All participants were informed about the purposes of the study and gave their informed consent in accordance with the Declaration of Helsinki (World Medical Association, 2013).

## Materials

### Individual differences questionnaires and test

#### Jigsaw puzzle test (JPT, De Beni et al., 2008)

This visuospatial working memory task consists of completing puzzles of increasing level of difficulties (i.e. from 2 to 10 pieces) without moving the pieces. Each difficulty level has two puzzles. Solving one correctly advances the task to the next level. The score is the sum of the levels of the last three correctly solved puzzles (max. 29). The reliability of the original version is good (r = 0.83).

#### Spatial anxiety scale (Lawton, 1994)

It consists of eight items regarding the degree of anxiety experienced in wayfinding situations (e.g., “Going to an appointment in an unfamiliar part of the city”) on a scale from 1 (not at all) to 6 (very much). The score is the sum of the answers (max 48). The questionnaire has good reliability (Cronbach’s alpha = 0.89 for the current sample).

#### Self-efficacy and pleasure in exploring scale (SEPE; created ad hoc)

It consists of 22 items regarding effectiveness and pleasure in exploration behaviors in everyday life (e.g., “I enjoy exploring new roads instead of retracing the ones that I already know”; see supplementary materials for the complete list of items). Participants are asked to express a degree of agreement on a scale from 1 = strongly disagree to 7 = strongly agree. The score is the sum of the item answers.

The validity of the questionnaire (originally 24 items long) was evaluated with a different sample of 372 people 18 to 60 years old (237 women), collected online (random presentation of items). We assessed the factorial structure of the scale, test re-test reliability (a part of the sample re-filled the questionnaire three weeks later) and convergent validity (with correlation of Santa Barbara Sense of Direction, a widely used and validated measure of self-reported navigation ability). Considering the structural factor, we treated the Likert scales of the items as ordinal variables using the Diagonally Weighted Least Squares (DWLS) estimator (e.g., Brauer et al., 2023) and we found barely adequate fit indexes for the model (RMSEA = 0.15, SRMR = 0.11, CFI = 0.93, TLI = 0.93, NNFI = 0.93). We inspected the modification indexes, and we refitted the model considering the items with higher residual correlations (modification indexes higher than 10). These residual correlations were theoretically interpretable, mainly among items with overlapping content or similar wording (see details in supplementary materials). After this correction, we found adequate fit indexes for the model (RMSEA = 0.048, SRMR = 0.048, CFI = 1.00, TLI = 0.99, NNFI = 0.99). Two items loaded < .40 on the factor therefore were eliminated from the questionnaire, after this correction the model indexes little increased (RMSEA = 0.048, SRMR = 0.046, CFI = 1.00, TLI = 0.99, NNFI = 0.99). The questionnaire had good reliability, Cronbach's alpha = 0.92, test re-test reliability (in a subsample of N = 104 participants, 50 women: *r* = .72) and good convergent validity (correlation with the SBSOD *r* = .73). The model was re-tested on the current sample, showing acceptable fit indices (RMSEA = 0.052, SRMR = 0.054, CFI = 1.00, TLI = 0.99, NNFI = 0.99).

#### Familiarity with the joystick question

It consists of a question assessing the participant level of familiarity with the joystick, using a scale from 1 (not at all) to 9 (extremely familiar).

#### Virtual reality exploration training phase

It consists of free exploration in a simplified virtual environment containing three-dimensional geometric shapes (a parallelepiped, a cube, and a sphere), presented in a CAVE lab setting. This ensures familiarity with joystick operation and the free exploration task. The training concludes when the participant reports feeling confident (after at least one minute).

#### City environment exploration phase

##### The environment

**Fig. 2:**
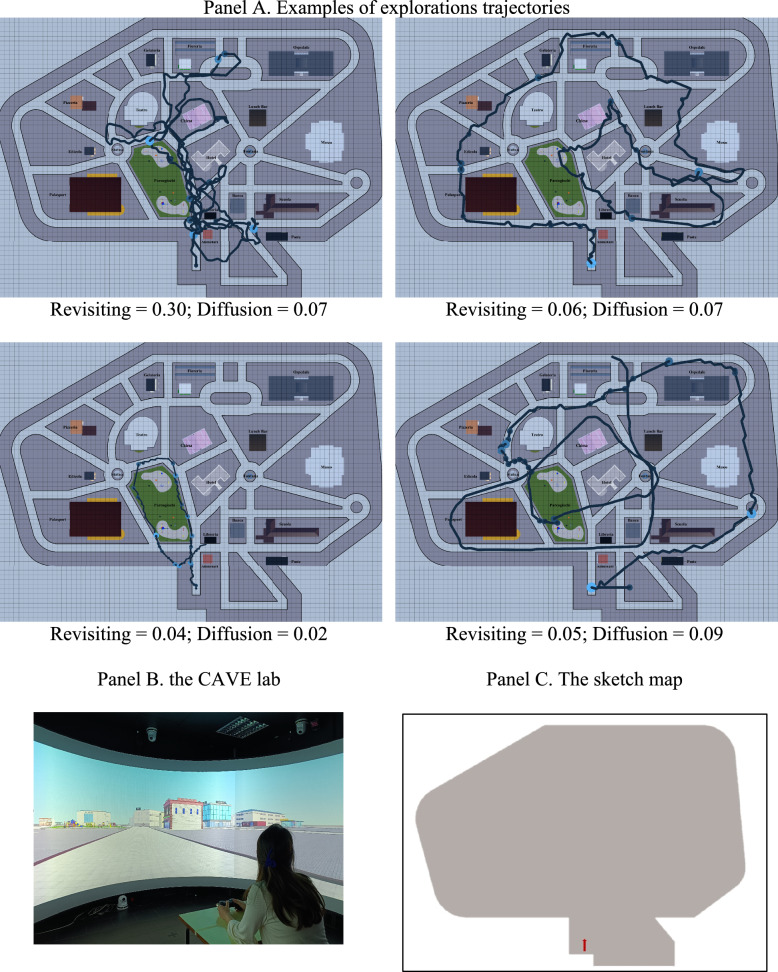
Examples of free exploration trajectories (panel **A**), the VR in the CAVE lab (panel **B**) and the sketch map used in the sketch map task (panel **C**). *Note*. Grid squares are 5 × 5 m

The environment consists of a virtual city measuring about 380 m x 270 m featuring 19 landmarks (bank, church, school, flower shop, fountain, grocery store, hospital, hotel, ice cream parlor, library, lunch bar, museum, newsstand, playground, pizzeria, post office, stadium, statue, theater) arranged within a grid of streets (Miola et al., 2021; Muffato et al., 2025). The virtual simulation is conducted within a CAVE lab environment (Fig. 2), where participants are positioned centrally in the room and immersed in projections spanning over 180°. It replicates stereoscopic vision using frame-sequential projection (resolution of 5760 × 1080 pixels at 60 Hz). The virtual environment was modelled using the Blender software, while data collection was performed using Godot v3.5.3. It was set a first-person perspective at a fixed height of 1.60 m and a movement speed of 7 m/s (speed selected based on pilot testing to allow comfortable exploration).

*The exploration phase.* It consists of a free navigation for five minutes in the virtual city in order to learn about the various elements of the city (buildings and streets) and their relative positions (see examples of trajectories in Fig. 2, panel A). To assess the free exploration behavior, revisiting and diffusion indexes were calculated by dividing the environment into 5-by-5-m squares (adapting the approach of Gagnon et al., 2016; see the grid of squares in Fig. 2, panel A). Revisiting was calculated as [1–(number of squares explored)/(number of segments traveled)], where a segment refers to a portion of path starting and ending within a single square. Therefore, a value of zero indicates that each segment falls within a new square, whereas increasing values indicate that multiple segments occur within the same square, reflecting a greater tendency to revisit previously explored areas. Diffusion was calculated as (number of squares explored) / (number of total squares), with higher values indicating a greater extent of environmental coverage during exploration. Since the data allowed, additional exploration indices were computed. Path roaming entropy, measured using the R entropy package, with higher values indicating that the participant explored the environment broadly and unpredictably, without favoring specific areas. The Pause number, representing any stop lasting more than 3 s, served as an indicator of uncertainty during exploration. Finally, the path length representing the total distance traveled, with higher values indicating longer distances.

#### Free sketch map task

It consists of drawing the landmarks and streets recalled on a blank map of the environment, provided only with borders and an arrow indicating the starting point of the exploration phase (Fig. 2, panel C). Sketch maps are analyzed using the Gardony Map Drawing Analyzer (GMDA, Gardony et al., 2016), and the square root of the canonical organization (SQRTCO; max. 1) is used as a measure of the global accuracy of landmark positioning.

### Procedure

The study consisted of three individual sessions that lasted approximately 45 min in total. The first session was conducted online through a Qualtrics link (20 min), while the second (10 min) and third (15 min) sessions took place in person in the labs. After signing the consent form, in the first session participants provided demographic data (gender, age) and in a balanced order performed the spatial anxiety scale and the self-efficacy and pleasure in exploring questionnaire. Two additional measures, not of interest in the present study, were collected: the Object-Spatial Imagery and Verbal Questionnaire (Blazhenkova & Kozhevnikov, 2009) and the Behavioral, Emotional, and Social Skills Inventory-I-45 (Feraco et al., 2024). See supplementary material for more information.

In the second session (on another day of the same week), the participants met the experimenter in a lab to complete the Jigsaw Puzzle Test. In the third session (on the same day as the second session), participants entered the CAVE lab. First, participants rated their familiarity with the joystick. Next, a training phase allowed participants to freely navigate a simplified virtual environment with three-dimensional objects, helping them become familiar with the movement and the free exploration task. Following this, the free exploration phase began, lasting five minutes for all participants (a duration selected based on pilot testing to allow comfortable exploration). They received the following instructions: "Now you will see a virtual city. Your task is to move freely within this city to learn about the environment. Pay close attention to the buildings and landmarks you encounter, which will be marked with clearly visible labels. Your goal is to familiarize yourself as much as possible with the elements and the environment in general. You are free to explore at your own pace and you will have all the time you need. When the exploration time ends, the task will stop automatically, so continue exploring until it concludes." All participants freely explored by moving the joystick until the task software disabled this function. After five minutes of free exploration, participants completed the sketch map task.

### Data analysis

We conducted analyses using R (R Core Team, 2024).

As a preliminary step, since our focus was on the revisiting and diffusion indexes, but data were also available for Shannon’s entropy, path length, and number of pauses, we examined the correlations among all these measures of exploration behavior. As another preliminary step, we examined gender differences between all variables considered, given that gender is an important factor typically associated with spatial knowledge and exploration behavior (e.g., Gagnon et al., 2016; Munion et al., 2019). See Supplementary.

For the main aim, we computed the means, standard deviations, and correlations of demographics (gender, age, joystick familiarity), visuospatial working memory (Jigsaw Puzzle Test), self-efficacy and pleasure in exploring, spatial anxiety, exploration behavior (revisiting and diffusion indexes), and survey knowledge (map drawing task) to provide descriptive statistics. To examine our hypotheses about the relationships among individual differences, exploration behaviors, and survey knowledge, we performed a structural equation analysis. Specifically, we tested (i) the relationship between individual differences and exploration behaviors, (ii) the relationship between individual differences and survey knowledge after exploration, and (iii) whether exploration behavior mediates the link between individual differences and survey knowledge. In this analysis, we considered survey knowledge (measured using the map drawing task) as the dependent variable. Individual differences, such as gender, age, joystick familiarity, visuospatial working memory, self-efficacy and pleasure in exploring, and spatial anxiety, were included as predictors. Exploration behaviors, in terms of the diffusion and revisiting indexes, were included as mediators, representing two distinct core measures of the exploration pattern, with a hypothesized negative relationship between them. We considered covariance between predictors and between the mediators, the model is a saturated model (i.e., no degrees of freedom; see the structure of the model in the supplementary materials). We reported standardized betas, CIs, and p-values, the variance explained by the mediators and the dependent variable of the model. We checked the VIF values and Cook’s distances (Cook, 1986). Finally, we reported the fit indices of the model after removing the insignificant relationships (Schreiber et al., 2006). We considered the root mean square error of the approximation (RMSEA, range 0–1), the standardized root mean square residual (SRMR, range 0–1), comparative fit index (CFI, range0–1), and the non-normed fit index (NNFI, range 0–1). To further corroborate the findings, we also ran five additional models including each single exploration index—revisiting, diffusion, entropy, path length, and number of pauses—as mediators (see supplementary materials).

## Results

### Relationship between individual differences, exploration behavior, and survey knowledge

At a descriptive level, see Table 1 for means and standard deviations of all considered variables and correlations between them.

**Table 1:** Descriptives and correlations between variables of interest

	Men *M(DS)*	Women *M(DS)*	1^+^	2	3	4	5	6	7	8
1. Gender^+^	/	/	−							
2. Age	21.70^#^(2.29)	20.77(1.48)	− 0.23	–						
3. Joystick familiarity	6.95^#^(2.03)	4.17(2.35)	− 0.53	0.15	–					
4. visuospatial working memory (Jigsaw Puzzle Test)	24.22^#^(3.99)	21.54(5.01)	− 0.28	0.01	0.29	–				
5. Spatial anxiety	17.90^#^(5.07)	21.48(5.77)	0.30	− 0.23	− 0.19	− 0.21	–			
6. Self-Efficacy and Pleasure in Exploring	101.33^#^(19.62)	89.77(22.16)	− 0.26	0.10	0.14	0.21	− 0.64	–		
7. Exploration behavior – Revisiting	0.20^#^(0.08)	0.17(0.08)	− 0.21	− 0.04	0.18	0.05	0.00	− 0.16	–	
8. Exploration behavior – Diffusion	0.07^#^(0.01)	0.06(0.01)	− 0.43	0.12	0.39	0.17	− 0.11	0.18	− 0.09	–
9. Survey knowledge – Map drawing accuracy	0.73^#^(0.15)	0.70(0.16)	− 0.09	0.03	0.02	0.22	− 0.09	0.09	0.21	− 0.15

Note. *N* = 234. |r|≥ 0.14, *p* < 0.05; |r|≥ 0.18, *p* < 0.01; |r|≥ 0.22, *p* < 0.001. ^+^Spearman correlations (1 = men, 2 = women). ^#^significant differences between women and men (t-tests, *ps* < 0.05)

**Fig. 3:**
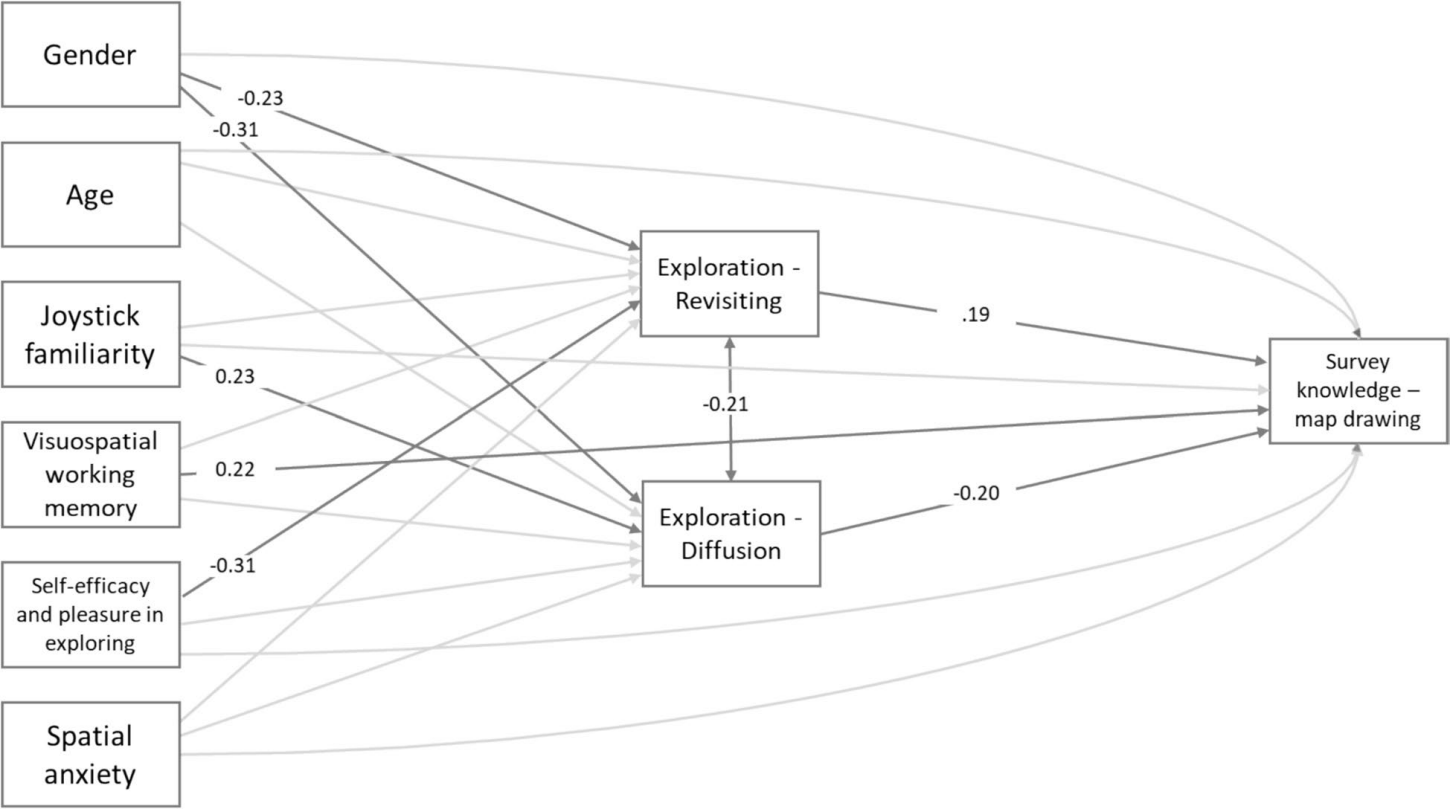
Graphical representation of the structural equation model. *Note*: Significant paths are shown in black. Covariances between predictors are accounted for in the model (saturated model) but are not displayed in the figure. Gender coding: 1 = men, 2 = women

Then, we computed the mediation (saturated) model. Standardized betas, confidence intervals (CI) and *p* values for the mediation model are presented in Table 2 (covariances are presented in Table S2), with a graphical representation provided in Fig. 3. Individual differences explained 12% of revisiting, 24% of diffusion, and 13% of map-drawing variance. No significant multicollinearity was found (VIF < 1.32), and Cook’s distances were < 1, except one case. Removing it slightly changed paths but not the results pattern, so the model with all observations was retained. To check the robustness of the model, we calculated confidence intervals using 5000 bootstrap resamples and found a similar pattern of results for the mediation of diffusion between gender and survey knowledge, as well as for the mediation of revisiting between self-efficacy and pleasure in exploring and survey knowledge. On the other hand, the indirect paths from gender through revisiting to survey knowledge and from joystick familiarity through diffusion to survey knowledge approached the critical *p* value of 0.05 therefore were not considered significant in the model (see Supplementary Materials and Table 2). The final model, after removing the insignificant relationships, has good fit indexes: RMSEA = 0.02, SRMR = 0.03, CFI = 0.99, NNFI = 0.99.

**Table 2:** Mediation model standardized betas, confidence interval and p values

			Std β	CI lower	CI upper	*p*
*Direct effects*						
Gender	** → **	**Exploration behavior -Revisiting**	− **0.23**	− **0.37**	− **0.08**	**0.002**
Age	→	Exploration behavior -Revisiting	− 0.11	− 0.23	0.02	0.089
Joystick familiarity	→	Exploration behavior -Revisiting	0.10	− 0.05	0.24	0.185
visuospatial working memory (Jigsaw Puzzle Test)	→	Exploration behavior -Revisiting	0.00	− 0.13	0.12	0.949
Self-Efficacy and Pleasure in Exploring	** → **	**Exploration behavior -Revisiting**	− **0.31**	− **0.46**	− **0.15**	** < 0.001**
Spatial anxiety	→	Exploration behavior -Revisiting	− 0.13	− 0.29	0.03	0.115
Gender	** → **	**Exploration behavior -Diffusion**	− **0.31**	− **0.45**	− **0.18**	** < 0.001**
Age	→	Exploration behavior -Diffusion	0.02	− 0.09	0.14	0.690
Joystick familiarity	** → **	**Exploration behavior -Diffusion**	**0.23**	**0.10**	**0.35**	**0.001**
Visuospatial working memory (Jigsaw Puzzle Test)	→	Exploration behavior -Diffusion	0.01	− 0.10	0.13	0.810
Self-Efficacy and Pleasure in Exploring	→	Exploration behavior -Diffusion	0.14	0.00	0.29	0.054
Spatial anxiety	→	Exploration behavior -Diffusion	0.13	− 0.02	0.28	0.097
Gender	→	Survey knowledge -map drawing task	− 0.08	− 0.23	0.08	0.339
Age	→	Survey knowledge -map drawing task	0.04	− 0.08	0.17	0.512
Joystick familiarity	→	Survey knowledge -map drawing task	− 0.06	− 0.20	0.09	0.445
Visuospatial working memory (Jigsaw Puzzle Test)	** → **	**Survey knowledge -map drawing task**	**0.22**	**0.10**	**0.35**	** < 0.001**
Self-Efficacy and Pleasure in Exploring	→	Survey knowledge -map drawing task	0.12	− 0.04	0.28	0.153
Spatial anxiety	→	Survey knowledge -map drawing task	0.03	− 0.13	0.19	0.711
Exploration behavior – Revisiting	** → **	**Survey knowledge -map drawing task**	**0.19**	**0.06**	**0.32**	**0.003**
Exploration behavior – Diffusion	** → **	**Survey knowledge -map drawing task**	− **0.20**	− **0.34**	− **0.06**	**0.004**
*Indirect effects*						
Gender → Revisiting → Survey knowledge			− 0.04	− 0.08	0.00	0.034
**Gender → Diffusion → Survey knowledge**			**0.06**	**0.01**	**0.11**	**0.015**
Age → Revisiting → Survey knowledge			− 0.02	− 0.05	0.01	0.143
Age → Diffusion → Survey knowledge			0.00	− 0.03	0.02	0.693
Joystick familiarity → Revisiting → Survey knowledge			0.02	− 0.01	0.05	0.229
Joystick familiarity → Diffusion → Survey knowledge			− 0.05	− 0.09	0.00	0.029
visuospatial working memory (Jigsaw Puzzle Test) → Revisiting → Survey knowledge			0.00	− 0.03	0.02	0.949
visuospatial working memory (Jigsaw Puzzle Test) → Diffusion → Survey knowledge			0.00	− 0.03	0.02	0.811
**Self-Efficacy and Pleasure in Exploring → Revisiting → Survey knowledge**			− **0.06**	− **0.11**	− **0.01**	**0.020**
Self-Efficacy and Pleasure in Exploring → Diffusion → Survey knowledge			− 0.03	− 0.06	0.01	0.111
Spatial anxiety → Revisiting → Survey knowledge			− 0.02	− 0.06	0.01	0.166
Spatial anxiety → Diffusion** → **Survey knowledge			− 0.03	− 0.06	0.01	0.152

Note. In bold significant relationship (after inspecting the bootstrap results, a more conservative significance threshold of *p* < 0.02 was applied)

Furthermore, we ran five single-mediator models, each including one exploration index (revisiting, diffusion, entropy, path length, or number of pauses), to further verify the relationships between exploration indexes and map-drawing accuracy. These analyses confirmed a positive relationship between revisiting and map-drawing accuracy, and negative relationships for diffusion, entropy, and path length, with no effect for the number of pauses. Gender showed a direct effect on all five exploration indexes. The SEPE effect was significant for revisiting, approached significance for diffusion, and was observed for entropy. Regarding indirect effects, the revisiting and diffusion models mirrored the combined model. Entropy showed a similar pattern of indirect paths to diffusion, while no indirect effects emerged for path length or number of pauses (see details in supplementary materials).

## Discussion

This study aimed to investigate how individual differences, including gender, visuospatial working memory, self-efficacy and pleasure in exploring, and spatial anxiety, relate to exploration behaviors and the acquisition of survey knowledge, testing the mediating role of exploration behaviors between individual differences and survey knowledge. Participants freely explored a virtual city while their exploration patterns (e.g., revisiting and diffusion indexes) and survey knowledge (i.e., map-drawing task) were assessed. Gender, age, and joystick familiarity were also assessed.

First, the findings on the exploration patterns revealed an association with survey knowledge, as tested by the map drawing task. For our participants, lower diffusion and greater revisiting were associated with higher survey knowledge as assessed through the map drawing task (this was also confirmed by lower entropy and path length being associated with higher map accuracy). This relationship was different from what has been reported in the literature. In fact, previous studies found no relationship between entropy (which is positively correlated with the diffusion index) and map drawing tasks (Brunec et al., 2023), or a positive relationship between higher revisiting and greater pointing errors (Brunec et al., 2023; Gagnon et al., 2016). One possible explanation for this discrepancy may lie in the specific way survey knowledge was measured in our study. We used a map drawing task that evaluates whether participants placed landmarks in their correct positions relative to one another on a map. This task is rarely used in the exploration behavior literature and has shown mixed or null effects in relation to exploration indices (e.g., Brunec et al., 2023; Cen et al., 2024), whereas most previous studies have focused on navigation success or pointing tasks (e.g., Gagnon et al., 2016). Nonetheless, the map drawing task is generally considered a core measure of survey knowledge (e.g., Muffato et al., 2023), which suggests that the nature of the task may have played a role in the observed relationship. In addition, the instructions given to participants may have had a role in their exploration behavior. Because they were explicitly instructed to learn the landmarks and their locations, they may have been encouraged to disperse their exploration less and revisit areas more frequently to achieve this goal. Furthermore, the environment consisted only of roads with clearly visible landmarks, which may have further affected their approach to the task. This context may have favored individuals who engaged in revisiting, as revisiting in this case was likely not inefficient wandering but rather reinspection of relevant areas, supporting better survey knowledge of the landmarks. Similarly, participants who exhibited lower diffusion may have benefited from a more focused and detailed learning of the landmarks’ positions. Taken together, these results suggest that the method used to evaluate survey knowledge (in this case, a map drawing task), the instructions provided to participants (the role of the goal), and the characteristics of the environment may all contribute to explaining the relationship between exploration behaviors and survey knowledge. These aspects deserve further systematic investigation in future studies. At a broader level, this aligns with the issue in spatial cognition that concerns how the type of spatial task and the specific spatial ability measured (Uttal et al., 2024) relate to the characteristics of the environment. These environmental variations have, in turn, contributed to the development of new classifications of spatial knowledge acquired through environmental navigation (van der Ham & Claessen, 2020).

Concerning the role of visuospatial working memory, a clear pattern emerged. It is a strong predictor of map drawing accuracy (survey knowledge), similar to its role for survey knowledge after learning a predetermined path (Labate et al., 2014; Muffato et al., 2025). Even after free exploration of the environment, visuospatial working memory remains a key factor in constructing a refined mental representation of the environment. Interestingly, however, it is not related to exploration behaviors, suggesting that individuals with greater visuospatial working memory capacity do not exhibit distinct exploration patterns. This novel insight suggests that this cognitive factor plays a specific role in supporting the acquisition of spatial knowledge, regardless of how it is acquired.

Concerning the role of self-reported self-efficacy and pleasure in exploring and spatial anxiety, the results showed a negative direct effect of self-efficacy and pleasure in exploring on revisiting behaviors, which, in turn, contributed to better map drawing accuracy (effect confirmed for entropy and smaller for diffusion). On the other hand, no significant relationship was found for spatial anxiety. This finding is consistent with previous studies, which often failed to find a relationship between spatial anxiety—but also sense of direction—and exploration behavior (e.g., Doner et al., 2023; Munion et al., 2019). However, in contrast to these previous findings, our results newly showed that self-efficacy and pleasure specifically related to exploration seem to be more reliable in relating to the actual exploration behavior than general inclination such as spatial anxiety or sense of direction. Our findings suggest that confidence and enjoyment in exploration, as self-reported in daily life, are connected to the objective and actual ways individuals navigate the (virtual) environment. These inclinations and attitudes promote the avoidance of previously visited areas, without excessive repetition, ultimately supporting the development of survey knowledge. Specifically, in our study, it seems that individuals with high self-efficacy and pleasure in exploring, more so than those with reduced spatial anxiety (Lawton et al., 1994), may exhibit a favourable exploration profile, which should be considered and nurtured. However, it is worth noting that self-efficacy and pleasure in exploring are inversely highly correlated with spatial anxiety, suggesting that the role of spatial anxiety may not be fully captured in the model but could still be relevant (e.g., Gagnon et al., 2018). Further studies on self-reported wayfinding inclinations are needed, given their potential insights.

Concerning gender, the results showed that women tended to engage less in both revisiting and diffusion patterns of exploration than men, aligning with previous findings (e.g., Farran et al., 2022; Gagnon et al., 2018; Munion et al., 2019). The pattern was confirmed by women showing lower entropy, shorter path lengths, and a higher number of pauses. These results reflect an established difference in exploration patterns between men and women. However, an unexpected pattern emerged from the mediation analysis: the negative association between gender and diffusion (i.e., women showing lower diffusion) and the negative association between diffusion and survey knowledge produced a positive indirect effect. That is, women achieved higher survey knowledge through lower diffusion. This result may suggest that, given the instructions and the characteristics of the environment, women benefited from strategies they tend to use more frequently, such as remembering specific landmarks along a route, which have been shown to provide an advantage over men (e.g., Dahmani et al., 2023). These findings highlight the need for further research on gender differences in exploration behaviors and their effects on survey knowledge.

Age and joystick familiarity were considered. No age effects emerged, likely due to the young sample. Greater joystick familiarity, reflecting gaming experience, was associated with broader diffusion (and higher entropy, longer path lengths, and fewer pauses), suggesting that skilled users explore more. However, this may have a negative effect on survey knowledge (a small, non-significant, effect that warrants further investigation). Gaming experience and joystick familiarity (Diersch & Wolbers, 2019; Ward et al., 2025; Yavuz et al., 2024) are challenge factors for VR use in spatial navigation learning, highlighting the need for caution in VR-based studies. Future research should investigate real-world exploration, which remains underexamined.

This study offered valuable insights but had limitations. First, the virtual city with clear landmarks may not capture real-world complexity. Future research should use environments with varying size and complexity, possibly within-subject. Second, instructions focused on learning landmarks, which may have shaped participants’ behavior toward more targeted exploration; different goals should be investigated. Third, the fixed five minutes time limit may also have affected exploration patterns. In this literature, exploration time varies considerably across studies, ranging from no time limits (e.g., Gehrke et al., 2018) to longer durations (e.g., Brunec et al., 2023) or just a few minutes (e.g., Farran et al., 2022), and this point needs to be systematically investigated. Free-exploration without time-limit may capture navigation-variability patterns in relation to individual variables. Fourth, relying on a single map task may not fully assess survey knowledge; multiple tasks could provide a more comprehensive evaluation (e.g., adding a pointing task). Fifth, familiarity with the joystick and devices should be assessed using a scale rather than a single item. Sixth, although our data indicate that the new SEPE measure has convergent validity (i.e., its association with SBSOD, which is considered conceptually close to self-efficacy; Miola et al., 2021), more comprehensive validation is still required. For example, future work should test the measure in larger and independent samples, and include indicators of discriminant validity (e.g., a general self-efficacy scale or other non-spatial belief measures), which were not included in our current validation sample. Finally, variables such as age (considered across the lifespan), gender and biological sex, and self-reported factors such as exploration self-efficacy should be further investigated in future studies, as their potential interactions may help explain individual differences in exploration behavior and survey knowledge (e.g., Cheng et al., 2022; Ham et al., 2021).

To conclude, this study expanded knowledge on spatial cognition and individual differences. It showed that people have exploration patterns linked to factors like gender, joystick use, and self-efficacy and pleasure in exploring. Exploration behaviors related to survey knowledge directly and by mediating some individual differences. Notably, visuospatial working memory was found to be crucial only for survey knowledge. These results suggest that accounting for individual differences in exploration may help optimize mental representations.

## Supplementary Information

Additional file1 (PDF 303 KB)

## Data Availability

The study was not preregistered. Materials are available on request. Data are available on OSF at https://osf.io/8evu2/overview.
