# Supplementary material for: “Freely explore this environment”: individual differences in exploration behavior and survey knowledge
Source: Cogn Res Princ Implic. 2025 Dec 19;10:86. doi: 10.1186/s41235-025-00696-5 (PMC12717347; doi:10.1186/s41235-025-00696-5)
Supplement: Supplementary file 1 — Additional file1 (PDF 303 KB) [file 41235_2025_696_MOESM1_ESM.pdf]

Supplementary material

1. Self-Efficacy and Pleasure in Exploring (SEPE) scale

1.1. List of items

1	When I see a new road, I avoid taking it because I don't know where it leads.
2	I enjoy finding new roads even to reach familiar and well- known places.
3	I don't like the idea of visiting faraway and unknown places because I might get lost
4	I feel uncomfortable if I have to reach a place in an unfamiliar city or place.
5	I prefer to vary the route to reach the same destinations, rather than always taking the same road
6	I don't like exploring and discovering new places when I am travelling or visiting a new city
7	I like to imagine what the view of a place would be like from above.
8	When returning from a certain place, I like to try alternative routes compared to the one I took on the way there.
9	I like to learn roads and routes so that I can easily retrace them later without having to consult maps or GPS.
10	I enjoy exploring new roads instead of retracing the ones that I already know
11	I feel able to reach the location of an appointment in an unfamiliar area of the city
12	I feel effective at finding the right way to get to my destination even in an environment that I know little about.
13	I don't feel able to indicate in which direction places are located in relation to the position in which I am
14	I feel effective at going back and finding the right way even after getting lost in an area that I know little about.
15	I don't feel effective at creating a mental map of the environment where I am.
16	I am able to understand my position by referring to the map of the environment where I am.
17	I don't feel capable of remembering landmarks and turns in the routes I take.
18	I feel effective at finding a shortcut even without using tools such as GPS or maps.
19	I don't feel effective at orienting myself in a new city or in an unfamiliar environment
20	I feel able to autonomously explore new areas of the city even without relying heavily on tools such as maps or GPS.
21	I don't feel able to easily decide the direction to take when I'm at a junction or intersection.
22	I feel effective at finding alternatives to the main road when it is blocked or too busy, even without consulting a map or GPS.

1.2. Specification on the CFA

Model refinement was guided by modification indices, which suggested several theoretically interpretable residual correlations. These were mainly among items sharing similar wording or overlapping content, and were included to improve model fit while preserving the theoretical integrity of the factor structure. For instance, items 5 ↔ 10 both concern enjoyment in varying routes and exploring alternative paths; similarly, items 2 ↔ 10 refer to pleasure in discovering new roads and exploring familiar areas differently; and items 2 ↔ 5 reflect a general preference for taking new or alternative routes to reach destinations.

2. Results

2.1. Preliminary analyses on exploration behavior

We calculated the means, standard deviations and correlations among various indexes of exploration behavior (i.e., revisiting, diffusion, number of pauses, path length and Shannon's entropy; see Table S1). The diffusion index was found to be strongly correlated with the number of pauses, path length, and entropy indexes, with absolute correlation values ranging from .79 to .98. Therefore, we confirmed the decision to use the revisiting and diffusion indices in the subsequent analyses. These two indexes were selected as refined measures that capture two key aspects of exploration behavior: the tendency to revisit previously seen locations and the tendency to diffusely explore the environment (e.g., Gagnon et al., 2016).

Then we examined gender differences between all variables. We found that men reported higher joystick familiarity and visuospatial working memory, lower spatial anxiety, and greater self-efficacy and pleasure in exploring. They also showed more revisiting and diffusion behaviors and higher map drawing accuracy (*ps* < 0.05). See the means and standard deviations in Table 1. Furthermore, in the training phase, women explored the environment more extensively, as indicated by a longer training path length ($M = 651.39$ m, $SD = 465.39$) compared to men ($M = 513.29$ m, $SD = 429.48$).

Table S1. Descriptives and correlations between exploration behavior indexes

	Women *M (DS)*	Men *M (DS)*	1	2	3	4
1. Number of pauses	9.62 (4.83)	4.71 (4.25)				
2. Path length	1332.32 (305.36)	1721.92 (347.22)	-0.88^{***}			
3. Shannon's entropy	5.53 (0.25)	5.73 (0.24)	-0.76^{***}	0.84^{***}		
4. Revisiting	0.17 (0.08)	0.20 (0.08)	-0.27^{***}	0.31^{***}	-0.19^{**}	
5. Diffusion	0.06 (0.01)	0.07 (0.01)	-0.79^{***}	0.90^{***}	0.98^{***}	-0.09

6. Survey knowledge – Map drawing accuracy	0.70 (0.16)	0.73 (0.15)	0.02	-0.07	-0.17**	0.21**	-0.15*

2.2. The mediation model

The model tested is the following:

```
model <- '
Revisiting ~ a1*Gender + a2*Age + a3*Joystick_familiarity + a4*VSWM_Jigsaw + a5*SEPE +
a6*Spatial_anxiety
Diffusion  ~ b1*Gender + b2*Age + b3*Joystick_familiarity + b4*VSWM_Jigsaw + b5*SEPE +
b6*Spatial_anxiety

SQRTCO      ~ c1*Gender + c2*Age + c3*Joystick_familiarity + c4*VSWM_Jigsaw + c5*SEPE +
c6*Spatial_anxiety + d1*Revisiting + d2*Diffusion

Revisiting ~~ Diffusion

i_gender_via_revisiting   := a1*d1
i_gender_via_diffusion    := b1*d2
i_age_via_revisiting      := a2*d1
i_age_via_diffusion       := b2*d2
i_joystick_via_revisiting := a3*d1
i_joystick_via_diffusion  := b3*d2
i_vswm_via_revisiting     := a4*d1
i_vswm_via_diffusion      := b4*d2
i_sepe_via_revisiting     := a5*d1
i_sepe_via_diffusion      := b5*d2
i_sa_via_revisiting       := a6*d1
i_sa_via_diffusion        := b6*d2
'
```

In table S2 the covariances of the mediation model.

Table S2. Covariances of the mediation model

<u>**Covariances**</u>			
Gender	~~	Age	-0.24
Gender	~~	Joystick familiarity	-0.52
Gender	~~	visuospatial working memory (Jigsaw Puzzle Test)	-0.27
Gender	~~	SEPE	-0.26
Gender	~~	Spatial anxiety	0.30
Age	~~	Joystick familiarity	0.15
Age	~~	Puzzle	0.01
Age	~~	Self-Efficacy and Pleasure in Exploring	0.10
Age	~~	Spatial anxiety	-0.23
Joystick familiarity	~~	Puzzle	0.29
Joystick familiarity	~~	Self-Efficacy and Pleasure in Exploring	0.14
Joystick familiarity	~~	Spatial anxiety	-0.19
visuospatial working memory (Jigsaw Puzzle Test)	~~	Self-Efficacy and Pleasure in Exploring	0.21
visuospatial working memory (Jigsaw Puzzle Test)	~~	Spatial anxiety	-0.21

Self-Efficacy and Pleasure in Exploring	~~	Spatial anxiety	-0.64
Revisiting	~~	Diffusion	-0.21

To verify the robustness of the indirect effects reported in the main model, we re-estimated the indirect paths using 5,000 bootstrap resamples. The overall pattern of results remained stable, however the indirect effects Gender → Diffusion → Survey knowledge and Joystick familiarity → Diffusion → Survey knowledge approached the critical p-value of 0.05 (see Table S3).

Table S3. Indirect effects with 5,000 bootstrap resamples

	Std β	CI lower	CI upper	*p*
Indirect effects				
Gender→ Revisiting→ Survey knowledge	-0.014	-0.034	-0.004	0.05
Gender→Diffusion→ Survey knowledge	0.021	0.005	0.045	0.04
Age → Revisiting→ Survey knowledge	-0.002	-0.006	0.000	0.23
Age →Diffusion→ Survey knowledge	0.000	-0.003	0.002	0.72
Joystick familiarity→ Revisiting→ Survey knowledge	0.001	0.000	0.004	0.30
Joystick familiarity→Diffusion→ Survey knowledge	-0.003	-0.007	-0.001	0.05
visuospatial working memory (Jigsaw Puzzle Test) → Revisiting→ Survey knowledge	0.000	-0.001	0.001	0.95
visuospatial working memory (Jigsaw Puzzle Test)→Diffusion→ Survey knowledge	0.000	-0.001	0.001	0.82
Self-Efficacy and Pleasure in Exploring → Revisiting→ Survey knowledge	0.001	-0.001	0.000	0.03
Self-Efficacy and Pleasure in Exploring →Diffusion→ Survey knowledge	0.000	-0.001	0.000	0.17
Spatial anxiety → Revisiting→ Survey knowledge	-0.001	-0.002	0.000	0.19
Spatial anxiety →Diffusion→ Survey knowledge	-0.001	-0.002	0.000	0.19

The model's robustness was also verified by testing two separate models including single mediators, Revisiting and Diffusion, which showed the same pattern of results (see Tables S4 and S5). Furthermore, additional single-mediator models were run for the other exploration measures, i.e., Shannon's entropy (Table S6), path length (Table S7), and number of pauses (Table S8). The model with Shannon's entropy yielded a pattern similar to that of Diffusion, further confirming the results for this exploration behavior. In contrast, path length and number of pauses did not mediate any of the individual differences in survey knowledge assessed through the map-drawing task.

Table S4. Single-mediator model with Revisiting

			Std β	CI lower	CI upper	*p*
**Direct effects**						
**Gender**	→	**Exploration behavior -Revisiting**	**-0.23**	**-0.37**	**-0.08**	**0.002**
Age	→	Exploration behavior -Revisiting	-0.11	-0.23	0.02	0.089
Joystick familiarity	→	Exploration behavior -Revisiting	0.10	-0.05	0.24	0.185
visuospatial working memory (Jigsaw Puzzle Test)	→	Exploration behavior -Revisiting	0.00	-0.13	0.12	0.949
**Self-Efficacy and Pleasure in Exploring**	→	**Exploration behavior -Revisiting**	**-0.31**	**-0.46**	**-0.15**	**<0.001**
Spatial anxiety	→	Exploration behavior -Revisiting	-0.13	-0.29	0.03	0.115
Gender	→	Survey knowledge -map drawing task	0.00	-0.16	0.15	0.954
Age	→	Survey knowledge -map drawing task	0.04	-0.09	0.17	0.524
Joystick familiarity	→	Survey knowledge -map drawing task	-0.11	-0.25	0.04	0.148
**visuospatial working memory (Jigsaw Puzzle Test)**	→	Survey knowledge **-map drawing task**	**0.22**	**0.09**	**0.34**	**0.001**
Self-Efficacy and Pleasure in Exploring	→	Survey knowledge -map drawing task	0.10	-0.06	0.26	0.228
Spatial anxiety	→	Survey knowledge -map drawing task	0.01	-0.15	0.17	0.903
**Exploration behavior – Revisiting**	→	**Survey knowledge -map drawing task**	**0.23**	**0.10**	**0.36**	**<0.001**
**Indirect effects**						
**Gender→ Revisiting→ Survey knowledge**			**-0.05**	**-0.10**	**-0.01**	**0.020**
Age → Revisiting→ Survey knowledge			-0.03	-0.06	0.01	0.126
Joystick familiarity→ Revisiting→ Survey knowledge			0.02	-0.01	0.06	0.215
visuospatial working memory (Jigsaw Puzzle Test) → Revisiting→ Survey knowledge			0.00	-0.03	0.03	0.949
**Self-Efficacy and Pleasure in Exploring → Revisiting→ Survey knowledge**			**-0.07**	**-0.12**	**-0.02**	**0.009**
Spatial anxiety → Revisiting→ Survey knowledge			-0.03	-0.07	0.01	0.150

Table S5. Single-mediator model with Diffusion

			Std β	CI lower	CI upper	*p*
**Direct effects**						
**Gender**	→	**Exploration behavior -Diffusion**	**-0.31**	**-0.45**	**-0.18**	**<0.001**
Age	→	Exploration behavior -Diffusion	0.02	-0.09	0.14	0.690
**Joystick familiarity**	→	**Exploration behavior -Diffusion**	**0.23**	**0.10**	**0.35**	**0.001**
visuospatial working memory (Jigsaw Puzzle Test)	→	Exploration behavior -Diffusion	0.01	-0.10	0.13	0.810
Self-Efficacy and Pleasure in Exploring	→	Exploration behavior -Diffusion	0.14	0.00	0.29	0.054
Spatial anxiety	→	Exploration behavior -Diffusion	0.13	-0.02	0.28	0.097
Gender	→	Survey knowledge -map drawing task	-0.13	-0.29	0.02	0.088

Age	→	Survey knowledge -map drawing task	0.02	-0.11	0.15	0.731
Joystick familiarity	→	Survey knowledge -map drawing task	-0.03	-0.18	0.12	0.703
**visuospatial working memory (Jigsaw Puzzle Test)**	→	Survey knowledge **-map drawing task**	**0.22**	**0.09**	**0.35**	**0.001**
Self-Efficacy and Pleasure in Exploring	→	Survey knowledge -map drawing task	0.06	-0.10	0.23	0.427
Spatial anxiety	→	Survey knowledge -map drawing task	0.01	-0.15	0.18	0.892
**Exploration behavior – Diffusion**	→	**Survey knowledge -map drawing task**	**-0.25**	**-0.38**	**-0.11**	**<0.001**
<u>**Indirect effects**</u>						
**Gender→ Diffusion→ Survey knowledge**			**0.08**	**0.02**	**0.13**	**0.005**
Age → Diffusion→ Survey knowledge			-0.01	-0.03	0.02	0.692
**Joystick familiarity→ Diffusion→ Survey knowledge**			**-0.06**	**-0.10**	**-0.01**	**0.015**
visuospatial working memory (Jigsaw Puzzle Test) → Diffusion→ Survey knowledge			0.00	-0.03	0.03	0.810
Self-Efficacy and Pleasure in Exploring → Diffusion→ Survey knowledge			-0.04	-0.08	0.01	0.092
Spatial anxiety → Diffusion→ Survey knowledge			-0.03	-0.07	0.01	0.134

Table S6. Single-mediator model with Shannon's entropy

			Std β	CI lower	CI upper	*p*
<u>**Direct effects**</u>						
**Gender**	→	**Exploration behavior -entropy**	**-0.25**	**-0.39**	**-0.11**	**<0.001**
Age	→	Exploration behavior -entropy	0.05	-0.07	0.17	0.405
**Joystick familiarity**	→	**Exploration behavior -entropy**	**0.22**	**0.08**	**0.35**	**<0.001**
visuospatial working memory (Jigsaw Puzzle Test)	→	Exploration behavior -entropy	0.03	-0.09	0.16	0.593
**Self-Efficacy and Pleasure in Exploring**	→	**Exploration behavior -entropy**	**0.16**	**0.01**	**0.31**	**0.038**
Spatial anxiety	→	Exploration behavior -entropy	0.15	0.00	0.30	0.056
Gender	→	Survey knowledge -map drawing task	-0.12	-0.27	0.03	0.116
Age	→	Survey knowledge -map drawing task	0.03	-0.10	0.16	0.647
Joystick familiarity	→	Survey knowledge -map drawing task	-0.03	-0.17	0.12	0.708
**visuospatial working memory (Jigsaw Puzzle Test)**	→	**Survey knowledge -map drawing task**	**0.23**	**0.10**	**0.35**	**0.001**
Self-Efficacy and Pleasure in Exploring	→	Survey knowledge -map drawing task	0.07	-0.09	0.23	0.385
Spatial anxiety	→	Survey knowledge -map drawing task	0.02	-0.15	0.18	0.821
**Exploration behavior – Entropy**	→	**Survey knowledge -map drawing task**	**-0.26**	**-0.39**	**-0.13**	**<0.001**
<u>**Indirect effects**</u>						
**Gender→ Entropy→ Survey knowledge**			**0.06**	**0.02**	**0.11**	**0.010**
Age → Entropy→ Survey knowledge			-0.01	-0.05	0.02	0.416
**Joystick familiarity→ Entropy→ Survey knowledge**			**-0.06**	**-0.10**	**-0.01**	**0.015**

visuospatial working memory (Jigsaw Puzzle Test) ⟶ Entropy⟶ Survey knowledge	-0.01	-0.04	0.02	0.597
Self-Efficacy and Pleasure in Exploring ⟶ Entropy⟶ Survey knowledge	-0.04	-0.08	0.00	0.069
Spatial anxiety ⟶ Entropy⟶ Survey knowledge	-0.04	-0.08	0.01	0.088

Table S7. Single-mediator model with path length

			Std β	CI lower	CI upper	*p*
<u>**Direct effects**</u>						
**Gender**	→	**Exploration behavior -path length**	**-0.40**	**-0.521**	**-0.277**	**<0.001**
**Age**	→	**Exploration behavior -path length**	**-0.04**	**-0.153**	**0.071**	**<0.001**
**Joystick familiarity**	→	**Exploration behavior -path length**	**0.25**	**0.127**	**0.373**	**<0.001**
visuospatial working memory (Jigsaw Puzzle Test)	⟶	Exploration behavior -path length	0.01	-0.102	0.127	0.828
Self-Efficacy and Pleasure in Exploring	⟶	Exploration behavior -path length	-0.02	-0.159	0.121	0.794
Spatial anxiety	⟶	Exploration behavior -path length	0.03	-0.111	0.176	0.656
Gender	⟶	Survey knowledge -map drawing task	-0.12	-0.279	0.045	0.158
Age	⟶	Survey knowledge -map drawing task	0.01	-0.120	0.141	0.875
Joystick familiarity	⟶	Survey knowledge -map drawing task	-0.05	-0.198	0.104	0.543
**visuospatial working memory (Jigsaw Puzzle Test)**	⟶	**Survey knowledge -map drawing task**	**0.22**	**0.091**	**0.347**	**0.001**
Self-Efficacy and Pleasure in Exploring	⟶	Survey knowledge -map drawing task	0.03	-0.135	0.189	0.742
Spatial anxiety	⟶	Survey knowledge -map drawing task	-0.01	-0.181	0.152	0.861
**Exploration behavior – Path length**	⟶	**Survey knowledge -map drawing task**	**-0.15**	**-0.296**	**-0.001**	**0.048**
<u>**Indirect effects**</u>						
Gender⟶ Path length⟶ Survey knowledge			0.06	0.00	0.12	0.060
Age ⟶ Path length⟶ Survey knowledge			0.01	-0.01	0.02	0.503
Joystick familiarity⟶ Path length⟶ Survey knowledge			-0.04	-0.08	0.00	0.077
visuospatial working memory (Jigsaw Puzzle Test) ⟶ Path length⟶ Survey knowledge			0.00	-0.02	0.02	0.829
Self-Efficacy and Pleasure in Exploring ⟶ Path length⟶ Survey knowledge			0.00	-0.02	0.02	0.796
Spatial anxiety ⟶ Path length⟶ Survey knowledge			0.00	0.00	0.12	0.060

Table S8. Single-mediator model with number of pauses

			Std β	CI lower	CI upper	*p*
<u>**Direct effects**</u>						
**Gender**	→	**Exploration behavior -pauses**	**0.42**	**0.29**	**0.55**	**<0.001**
Age	→	Exploration behavior -pauses	0.08	-0.03	0.20	0.163
**Joystick familiarity**	→	**Exploration behavior -pauses**	**-0.15**	**-0.28**	**-0.01**	**0.030**

visuospatial working memory (Jigsaw Puzzle Test)	→	Exploration behavior -pauses	0.00	-0.12	0.12	0.996
Self-Efficacy and Pleasure in Exploring	→	Exploration behavior -pauses	0.01	-0.14	0.15	0.934
Spatial anxiety	→	Exploration behavior -pauses	-0.04	-0.19	0.11	0.637
Gender	→	Survey knowledge -map drawing task	-0.06	-0.23	0.10	0.467
Age	→	Survey knowledge -map drawing task	0.02	-0.12	0.15	0.813
Joystick familiarity	→	Survey knowledge -map drawing task	-0.08	-0.23	0.07	0.276
**visuospatial working memory (Jigsaw Puzzle Test)**	→	**Survey knowledge -map drawing task**	**0.22**	**0.09**	**0.35**	**0.001**
Self-Efficacy and Pleasure in Exploring	→	Survey knowledge -map drawing task	0.03	-0.13	0.19	0.719
Spatial anxiety	→	Survey knowledge -map drawing task	-0.02	-0.19	0.15	0.821
Exploration behavior – Pauses	→	Survey knowledge -map drawing task	0.01	-0.13	0.15	0.910
<u>**Indirect effects**</u>						
Gender→ Pauses→ Survey knowledge			0.003	-0.06	0.06	0.910
Age → Pauses→ Survey knowledge			0.001	-0.01	0.01	0.910
Joystick familiarity→ Pauses→ Survey knowledge			-0.001	-0.02	0.02	0.910
visuospatial working memory (Jigsaw Puzzle Test) → Pauses→ Survey knowledge			0.000	0.00	0.00	0.996
Self-Efficacy and Pleasure in Exploring → Pauses→ Survey knowledge			0.000	0.00	0.00	0.947
Spatial anxiety → Pauses→ Survey knowledge			0.000	-0.01	0.01	0.913

3. The role of soft skills and cognitive styles

Our sample completed two additional questionnaires: (1) a soft skills questionnaire, which assesses social, emotional, and behavioral skills (BESSI; Soto et al., 2020; Italian version by Feraco et al., 2024) and (2) a cognitive styles questionnaire, which measures individual preferences in visual, spatial, and verbal processing (Blazhenkova & Kozhevnikov, 2009). We explored whether these personality-based factors were correlated with exploration behaviors and survey knowledge.

See the correlations in Table S9.

Table S9. Correlations between soft skills, cognitive styles and exploration behaviors and map drawing task

	BESSI self-management	BESSI innovation	BESSI cooperation	BESSI interaction	BESSI emotional regulation	OSIVQ Spatial style	OSIVQ Verbal style	OSIVQ Visual style
Number of pauses	0.11	-0.07	0.03	-0.06	**-0.15**	-0.08	**-0.14**	0.06
Revisiting	-0.09	-0.03	-0.10	-0.04	-0.02	-0.03	0.09	-0.11
Diffusion	-0.11	0.09	0.07	0.09	**0.21****	**0.16**	**0.15**	0.03
Map drawing task	-0.05	-0.10	-0.10	-0.08	0.01	0.09	0.01	-0.04

The correlations showed that emotional regulation (one of emotional skills; Soto et al., 2022) was positively associated with diffusion behavior and linked to fewer pauses. These findings align with previous research that highlighted the connection between emotions and spatial navigation (e.g., Ruotolo et al., 2019). Regarding cognitive style (Blazhenkova & Kozhevnikov, 2009), both verbal and spatial cognitive styles were correlated with diffusion behavior, with verbal styles also linked to fewer pauses. This suggests that preferred cognitive styles may structure how individuals decide to explore. However, these personality-related factors appear to be related to exploration behaviors without directly impacting spatial knowledge, as no correlation was found with the map-drawing task. This paves the way for a more systematic analysis of personality-related factors in exploration patterns.
